# The HAMP Signal Relay Domain Adopts Multiple Conformational States through Collective Piston and Tilt Motions

**DOI:** 10.1371/journal.pcbi.1002913

**Published:** 2013-02-28

**Authors:** Lizhe Zhu, Peter G. Bolhuis, Jocelyne Vreede

**Affiliations:** Van 't Hoff Institute for Molecular Sciences, University of Amsterdam, Amsterdam, The Netherlands; UNC Charlotte, United States of America

## Abstract

The HAMP domain is a linker region in prokaryotic sensor proteins and relays input signals to the transmitter domain and vice versa. Functional as a dimer, the structure of HAMP shows a parallel coiled-coil motif comprising four helices. To date, it is unclear how HAMP can relay signals from one domain to another, although several models exist. In this work, we use molecular simulation to test the hypothesis that HAMP adopts different conformations, one of which represents an active, signal-relaying configuration, and another an inactive, resting state. We first performed molecular dynamics simulation on the prototype HAMP domain Af1503 from *Archaeoglobus fulgidus*. We explored its conformational space by taking the structure of the A291F mutant disabling HAMP activity as a starting point. These simulations revealed additional conformational states that differ in the tilt angles between the helices as well as the relative piston shifts of the helices relative to each other. By enhancing the sampling in a metadynamics set up, we investigated three mechanistic models for HAMP signal transduction. Our results indicate that HAMP can access additional conformational states characterized by piston motion. Furthermore, the piston motion of the N-terminal helix of one monomer is directly correlated with the opposite piston motion of the C-terminal helix of the other monomer. The change in piston motion is accompanied by a change in tilt angle between the monomers, thus revealing that HAMP exhibits a collective motion, *i.e.* a combination of changes in tilt angles and a piston-like displacement. Our results provide insights into the conformational changes that underlie the signaling mechanism involving HAMP.

## Author Summary

For survival, bacteria must constantly monitor their environmental conditions and adapt to these by generating a response. Protein sensors enable bacteria to perceive their surroundings and are typically built from modular compounds that are connected by linker regions. The HAMP domain is such a linker region that relays signals between different modules in a sensory cascade. HAMP is a dimer comprising four helices in a parallel coiled-coil interaction motif. One of the hypotheses explaining the mechanism of signal communication by HAMP is that the domain can adopt different stable conformations. In this work, we used a molecular simulation approach to investigate this hypothesis at high atomic resolution. We found that HAMP can adopt different conformations and that, in doing so, the helices shift and tilt with respect to each other. Furthermore, we found that if one helix moves upward, the helix at the other end in the other monomer moves down.

## Introduction

**Figure 1:**
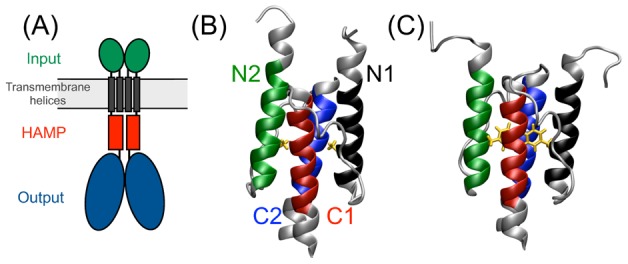
Position and structures of a canonical HAMP domain in the signaling complex. (A) Schematics of canonical HAMP connected to input and output domain; (B) Side view of the structure of wild-type Af1503-HAMP, (C) Side view of the A291F variant. The color code indicates the helix: *black* - N1, N-terminal helix of Monomer 1; *red* - C1, C-terminal helix of Monomer 1; *green* - N2, N-terminal helix of Monomer 2; *blue* - C2, C-terminal helix of Monomer 2; *orange* - residues with mutation; Colored helical residues are used in the calculation of the helical RMSD and helical properties.

To survive, bacteria must constantly monitor their environmental conditions and adapt to these by generating a response, such as a change in gene expression or motility. In bacteria, signaling proteins are built from modular components that regulate input, output and protein-protein communication. Many signaling proteins contain characteristic transmitter and receiver domains that promote information transfer within and between proteins. Signaling pathways are assembled by arranging these domains in various configurations [1], of which the simplest have two protein components: a sensor monitoring an environmental parameter, often located close to the membrane, and a cytoplasmic response regulator that mediates an adaptive response (*i.e.* a change in gene expression). The sensor typically contains an N-terminal input domain coupled to a C-terminal transmitter module. In many two-component signaling pathways, transmembrane 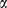-helices position the sensor/transmitter at the periplasmic side of the membrane, with the transmitter oriented toward the cytoplasm, see FIG. 1-A. Communication with the transmitter domain occurs via stimulus-induced conformational changes of the linker regions.

A typical linker region is the HAMP domain, originally identified in Histidine kinases, Adenylyl cyclases, Methyl-accepting chemotaxis protein and Phosphatases [2]. This 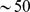 residue motif functions as a signal relay, converting the signal received into activation of the transmitter domain [3]. HAMP sequences contain heptad repeats (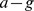), in which residues 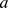 and 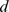 are typically hydrophobic, indicating that HAMP forms a coiled-coil complex. HAMP domains exist as a single unit, known as the the canonical form [3], but also occur in a sequentially repetitive fashion, known as the diverse form [4]–[6]. In the canonical form, the domain can be coupled to many different types of receptors and output regulators, such as diguanylate cyclases and phosphodiesterases [4]. As a repetitive domain, HAMP occurs both in intracellular signaling proteins [5] and transmembrane receptors [6]. Their wide occurrence, yet high structural similarity, may indicate a versatile mechanism for signal propagation in prokaryotes.

The first structure of a HAMP domain was resolved by NMR spectroscopy from the cytoplasmic *C*-terminal domain of the non-signaling trans-membrane (TM) protein Af1503 from the highly thermophilic organism *A. fulgidus* (PDB code 2L7H) [3]. Identification of this protein domain occurred through sequence similarity to known HAMP sequences [3], [7]. While lacking the periplasmic input and cytoplasmic output domains typically coupled to a canonical HAMP, the AF1503-HAMP domain shows activity when expressed in *E. coli*, substituting for the original HAMP domain in the chemotactic receptor Tar [8]–[10]. The structure of Af1503-HAMP shows a dimeric coiled-coil complex comprising four helices in a parallel orientation, shown in FIG. 1-B. The two monomers are labeled 1 and 2. Containing 58 residues, each monomer consists of two helices, labeled N and C, connected by a 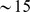 residue linker. The hydrophobic core of a canonical coiled coil comprises layers of residues 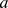 and 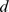 in the same heptad repeat, referred to as knobs-into-holes or (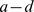) packing. Instead, the Af1503-HAMP structure displays an unusual packing in which each layer consists of either residues 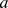 or 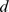 in the N-helices interacting with residues 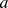 or 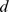 in the C-helices, see FIG. 1-C. As each helix contains two heptad repeats, the hydrophobic core of HAMP contains four layers. Additional residues directly preceding the 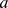-residues in the C-helices, or directly following the 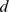-residues in the N-helices, contribute to the packing, and are therefore labeled 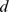 or 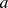 respectively. The residues in the helices that do not have neighboring residues contributing to the packing are labeled 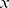. This packing is therefore referred to as complementary 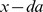 packing. The structure of Af1503-HAMP currently serves as the prototype structure of HAMP [3], [11].

HAMP functions as a signal relay domain between input and output domains of many bacterial sensor proteins, transmitting signals via conformational changes. An extensive mutagenesis study on the HAMP domain of the Tsr chemotaxis receptor provided insights into the mechanism of signal transduction by HAMP [12], [13] resulting in the dynamic bundle model. In this model, HAMP signal transduction occurs through changes in the stability of the helical bundle, modulated by conformational changes in the linker connecting HAMP to the transmembrane helices or changes in the stability of the output domain [12]. More importantly, the changes in stability of HAMP, induced by either input or output signals, cover a wide range of different conformations, indicating that HAMP function is more complex than an on-off switch [13].

Several models exist to describe the functional motions involved in the signal transduction mechanism of HAMP, including the gearbox model [3], the piston model [14]–[16] and a model describing helical tilting [11], [17]. Hulko et al. compared the complementary 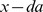 packing mode of the prototype structure and the knobs-into-holes packing of a typical coiled-coil structure, showing that a concerted helix rotation by 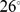 would convert the 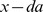 conformation into the canonical 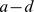 packing [3]. Ala291 in the prototype structure is an 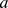-residue in the second heptad repeat of the N-helix and contributes to the packing as an 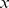-residue. Because small residues favor 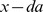 packing and large residues favor 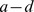 packing [18], residue 291 of Af1503-HAMP was changed to explore the influence of the sidechain size on adenylyl cyclase activity, using a chimeric assay system [3]. This mutation study revealed an inverse dependence of the activity on the volume of the hydrophobic sidechain at position 291 [3]. In particular, the A291V mutant reduced the activity to 62% compared to the wild type (WT) system and appeared to oscillate rapidly between two forms with presumably the 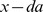 packing and the 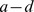 packing. Recently, structural data for most of these mutants became available [7], [19], revealing that there are several intermediate structures in the conversion between complementary 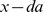 and knobs-into-holes packing modes [7]. The mutant A291F shows the highest structural diversity, as its crystal structure revealed an anti-parallel conformation, whereas in solution the mutant conformation is a mixture of parallel and anti-parallel conformations. The parallel conformation revealed the knobs-into-holes packing [7] with the corresponding helical rotation. Further evidence for helical rotation comes from the photoreceptor NpHtrII from *N. pharaonis*. Upon excitation by light, the NpHtrII transmembrane helices perform a 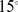 rotation and a displacement lateral to the membrane, as shown by electron paramagnetic resonance studies [20].

A second model is known as the piston model. Structural investigations on the aspartate chemoreceptor Tar in *E. coli* have shown that a transmembrane helix linked to a HAMP domain exhibits a piston-like motion inward to the cytoplasm upon binding of a signaling molecule to the periplasmic sensor domain [14], [15]. As the HAMP domain is directly connected to the transmembrane helix undergoing this inward motion, a piston-shift motion may play a role in HAMP mediated signal transduction. A mutation study focusing on positioning the transmembrane helix directly preceding the HAMP domain in the Tar receptor further confirmed that these helices exhibit a piston-like motion, inward to the cytoplasm [21]. Furthermore, a molecular simulation study looking into the position of the anchoring residues in the transmembrane helix of a chemotaxis receptor showed that downstream signaling activity was strongly correlated with a piston shift of 1.5 Å of the transmembrane helix [16].

In Ref. [11], Falke et al. confirmed the NMR structure of Af1503-HAMP as a structural template for the Tar HAMP domain and proposed, based on activity studies of Tar, a pivot model in which an initial piston motion may be able to tilt the helices from different subunits of HAMP with respect to each other. Helical tilting is also proposed as a model for signal relay based on in vivo cross-linking studies of a HAMP domain in the membrane based Aer sensor monitoring the intracellular redox potential [17]. Interestingly, this study found that the N-terminal helix in one monomer tilts in concert with the C-terminal helix in the other monomer.

Molecular simulation can complement experiments by modeling the dynamical time evolution of biomolecular systems in atomistic detail. A recent molecular dynamics study using a structural model of part of the Tar chemotaxis receptor elucidated the role of the connection between the transmembrane helices and HAMP in transmitting the signal from the sensor domain [22]. These simulations showed that HAMP exhibits larger fluctuations and a helical tilt upon a downward piston shift of the second transmembrane helix [22]. In this work, we aim to elucidate the nature of the signal transduction mechanism by HAMP, by investigating its equilibrium behavior via molecular dynamics (MD). In particular, we test the hypothesis that HAMP can adopt different conformations, of which one represents an active, signal-relaying configuration, and another an inactive, resting state. To this end, we perform regular MD simulations on Af1503-HAMP in two conformations. One conformation is the NMR structure, whereas an alternative conformation originated from the mutant A291F, which has a distinctly different packing. In addition, we enhance the sampling with metadynamics, which applies adaptive biasing potentials in MD simulations, based on predefined collective variables (CVs) [23]. These CVs constitute the motions the helices in HAMP exhibit with respect to each other: tilting, piston shift and rotation, based on the various models for the mechanism through which signals are relayed from the input domain, via HAMP, to an output domain. We find that Af1503-HAMP can adopt three additional conformational states besides the NMR structure, and that these states can inter-convert via changes in the piston shift of the helices. These conformational changes also directly lead to changes in the tilt angle between two HAMP monomers. Finally, biasing the helical rotation does not lead to a significant conformational change. This work supports the hypothesis that piston motions of the input domains connected to HAMP trigger the activation of HAMP by inducing piston motion in the output domain, most likely in combination with a tilting of the output domain helices.

## Results

### Af1503-HAMP is stable in solution

**Figure 2:**
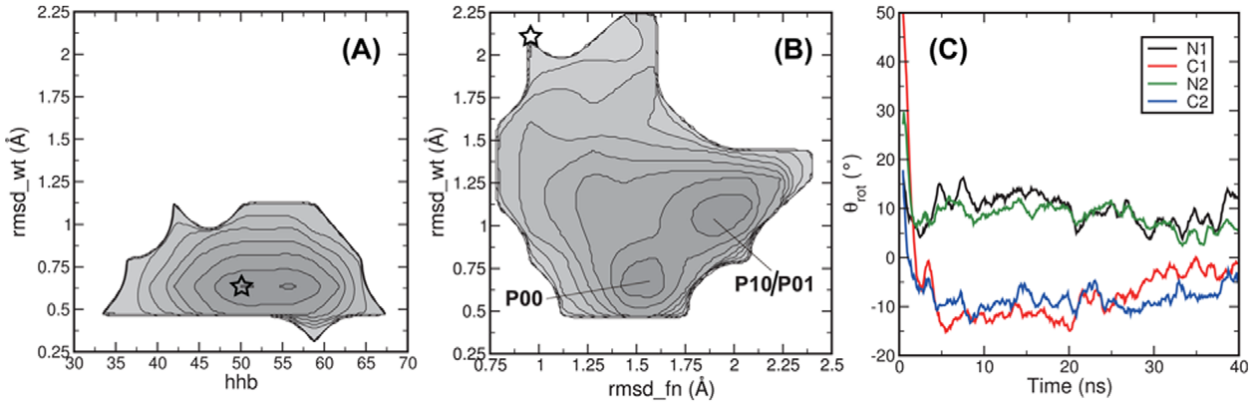
RMSD from experimental structures and helical rotation. The negative log of the probability distributions are shown for (A) the WT system as a function of the number of helical hydrogen bonds 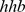 and the RMSD with respect to the NMR structure of WT, 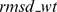 and (B) the WT* system as a function of the RMSD with respect to the NMR structure of the A291F mutant, 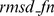 and 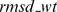. Labels indicate stable states. Contour lines are rendered every 1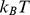. The stars indicate the starting point of the simulations. (C) Time evolution of helical rotation in the WT* system. The helical rotation for each helix is plotted as a function of simulation time, as a running average of 10 ps for a typical WT* simulation. The helical rotation is calculated as the angle between a reference point on the helix, the center of mass of the helix and the reference point on an aligned reference structure, see Methods for details. The MD simulations show that HAMP can visit additional conformational states.

Mutagenesis studies have shown that Af1503-HAMP has reduced activity upon altering the alanine at position 291. Increasing the volume of the hydrophobic sidechain at this position changes the packing in the hydrophobic packing from complementary 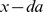 to 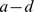 (knobs-into-holes) [7]. In this section, we perform MD simulations on wild-type and the mutant A291F Af1503-HAMP domains, to investigate the differences in structure and dynamics of these conformations. First we performed four 40 ns and four 60 ns MD simulations of the wild-type Af1503-HAMP domain, called WT hereafter, using the NMR structure (PDB code 2ASW [3]) as a starting point. Visual inspection revealed no dissociation of the complex or unfolding of the 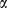-helical regions. As a quantitative measure we calculated the RMSD of the helices with respect to the NMR structure, 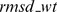, and the number of helical hydrogen bonds, 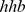, shown in FIG. 2-A as a contour plot of the negative natural logarithm of the probability distribution of these two measures. The profile displays a single minimum at 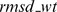 = 0.7 Å and 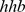 around 50. In other representations, including the helical rotation, 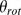, the inter-helical tilt angles 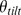, and the helical piston motion 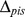, the WT simulations also display a single minimum. The values of these collective variables are listed in TAB. 1. The helices within one monomer have a tilt angle 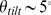 with respect to each other, while tilting angles between monomers are around 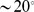. These angles are consistent with typical values observed in Ref. [3] and reflect that the monomers are not exactly parallel, but have a tilted orientation with respect to each other. Consequently, the HAMP domain resembles a cone with the tip at the C-terminal side, see FIG. 1-B. Finally, the helical piston shift in the WT system is very small. All these observations indicate that the structure resolved by NMR for the Af1503 HAMP domain is very stable as a single unit at room temperature.

**Table 1:** Averages of properties from MD simulations.

	WT Af1503
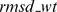 (Å)	0.712 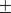 0.079
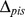 -M1AS1 (Å)	−0.020 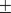 0.158
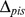 -M1AS2 (Å)	−0.044 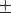 0.155
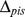 -M2AS1 (Å)	0.008 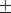 0.170
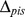 -M2AS2 (Å)	−0.021 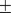 0.138
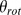 -M1AS1 (  )	6.70 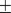 3.34
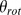 -M1AS2 (  )	−5.14 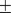 3.65
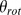 -M2AS1 (  )	4.74 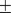 3.08
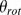 -M2AS2 (  )	−5.26 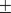 3.82
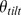 -M1AS1-M1AS2 (  )	4.82 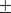 1.91
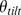 -M1AS1-M2AS1 (  )	21.1 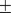 1.96
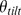 -M1AS1-M2AS2 (  )	20.3 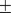 1.77
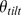 -M1AS2-M2AS1 (  )	20.1 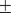 1.80
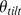 -M1AS2-M2AS2 (  )	20.9 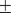 1.81
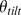 -M2AS1-M2AS2 (  )	6.94 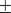 1.99
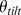 -M1-M2 (  )	18.1 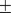 1.55

Properties: (I) 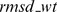; (II) piston motion of helices (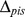); (III) helical rotation (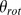); (IV) tilt angles between two helices (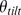). For Helical RMSD, rotation angles, and piston of helices, all frames are referenced on the NMR structure of WT Af1503.

### Relaxation from a perturbed structure reveals an additional conformational state

By increasing the volume of the hydrophobic sidechain at position 291, Ferris et al. have shown that the hydrophobic core can exhibit different packing modes that are in between the complementary 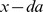 packing and canonical 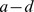 packing [7]. The A291F variant can adopt several conformations, including the 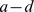 packing, as shown by NMR spectroscopy [7]. We performed MD simulations of this mutant, revealing that the parallel A291F structure is not stable in solution, see FIG. S1 in Text S1 for details. The simulations showed either the onset of helical unfolding or relaxation to a conformation obtained by fusing the A291F variant to a C-terminal domain [7]. We used this perturbed structure as a starting point to explore further the conformational space of the wild-type Af1503-HAMP. We therefore prepared a structure in which positions of atoms are identical to the NMR structure of the A291F mutant but with the phenylalanines on position 291 changed to alanines, again yielding the wild-type sequence. We performed 24 independent 50ns MD trajectories on this system, denoted as WT*. Most of these trajectories relax to 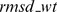 values of 1.2 Å or lower. In one out of the 24 trajectories, the helical bundle changes to an “out-of-register” conformation with a mismatch of the hydrophobic layers. This shifted register could be the result of a piston motion induced by asymmetric input from the sensor domains, pushing monomer 1 down with respect to monomer 2. However, there are several reasons to consider this conformation as misfolded rather than an alternative functional state of HAMP. As already noted in Refs. [11], [14], a register shift is too severe a change for a HAMP domain: the functional states of HAMP should closely resemble the Af1503-HAMP structure with only minor rearrangements [11], [14]. An out-of-register shift of the hydrophobic layers reflects a piston shift of 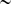
4–5 Å, which is larger than 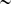
2 Å determined from crystallography studies of the input domain [14]. We therefore excluded this trajectory from further analysis.

FIG. 2-B displays the probability distribution as a function of 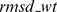 and the RMSD of the helices with respect to the NMR structure of the A291F mutant, 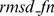, revealing two minima, 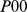 and 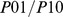. The minimum 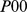 is identical to the configurations sampled in the WT-labeled simulations, as indicated by the low value for 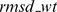. The minimum 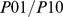 deviates from the wild-type configuration, but is also different from the A291F conformation. Note that this graph only gives an indication of the low free energy regions. In the WT* simulations, transitions from 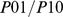 to 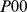 or vice versa occur only once in eight of the trajectories and not at all in the others, which is insufficient to give an accurate estimate of the free energy barriers separating the different states.

The simulations clearly show a relaxation from the A291F mutant structure with 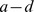 packing to conformations close to the structure of wild-type Af1503-HAMP, which may involve helical rotation, as postulated in the gearbox model [3]. The rotation of a helix along its principal axis can be defined in different ways. The program samCC can calculate several properties of helical bundles, including the Crick angle of a coiled-coil complex [4]. The Crick angle is defined for each residue as the angle between the center of the bundle, the residue and the center of the helix. This gives a measure for the rotation per residue. Instead, we computed the rotation of the entire helix, treated as a single rigid body, by defining a rotational reference point on the helix and then calculate the angle between this reference point on a structure, the helical center of mass and a reference structure: the NMR structure of wild-type Af1503-HAMP. This procedure is explained in detail in the Methods section. In FIG. 2-C, we plot the time evolution of the four helical rotation angles of a single, typical WT* simulation, which ends in the 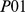 state. All helices start out with positive rotation values, an effect of aligning the conformation to a reference structure. The rotation angles drop to zero after a few ns, indicating the fast relaxation to conformations similar to wild-type Af1530 HAMP. During the fast relaxation, visual inspection revealed that the pairs of N and C-helices exhibit similar rotation, whereas an N-C pair rotate in opposite directions, in agreement with the gearbox model. Upon reaching the 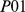 state, the N-helices have rotation angles of 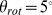 and the C-helices fluctuate around 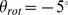, with respect to the reference structure. Visual inspection of the trajectories show that piston and tilting motions contribute to the relaxation process. The necessity of such motions can also be deduced from comparing the conformations of the wild-type HAMP and the A291F variant. As the two conformations have different bundle shapes, conversion of one into the other will require tilting of the helices and piston shifts to realign the hydrophobic layers.

**Figure 3:**
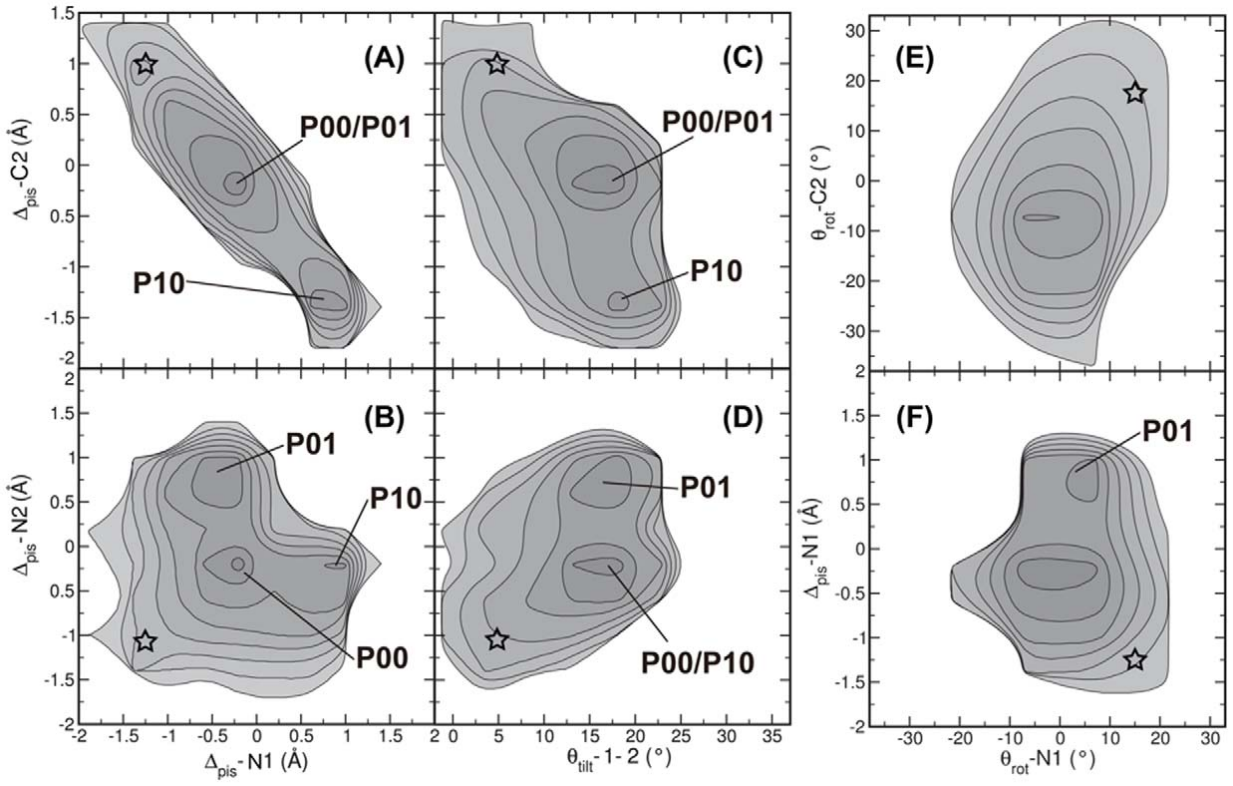
Piston shift, tilting and rotation of helices in WT* trajectories. The negative log of the probability distributions are plotted for (A) 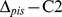 versus 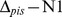; (B) 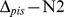 versus 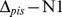; (C) 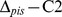 versus 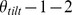; (D) 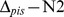 versus 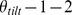; (E) 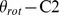 versus 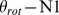; (F) 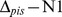 versus 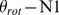. The labels indicate stable states. Contour lines are rendered every 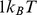. The stars indicate the starting point of the simulations. The WT* simulations reveal that the different conformational states of HAMP can be distinguished by differences in piston shift and tilt angle.

**Figure 4:**
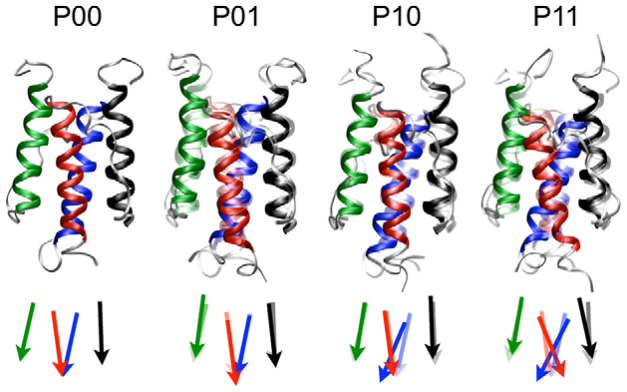
The four states of HAMP: *P*00, *P*10, *P*01, *P*11. Ribbon representations and schematic representations of the four states, with *black* N1; *red* C1; *green* N2; *blue* C2. Conformation 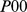 is rendered transparent in the depictions of states 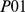, 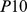 and 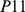 to illustrate the differences.

The new conformations in 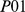/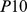 differ from the WT conformation in the values for the piston shifts, as shown in FIG. 3-A,B. FIG. 4 shows a schematic representation of the piston-shifted states. 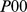 indicates the conformations close to WT, without any piston shifts; 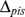 = 0 Å. The new conformational state 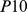/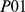 is split up in two symmetrically related states. Focusing on 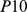, this state exhibits an upward piston shift of 1 Å for the N-helix in monomer 1 (N1), and a downward piston motion of 1.5 Å of the C-helix in monomer 2 (C2). Similarly, state 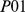 reveals a downshift of C1 in combination with an upshift of N2. We show all possible piston combinations in FIG. S2 in Text S1. The piston shifts fall within the range of 1–2 Å, as experimentally determined [14]. Strikingly, a piston shift of N1 is not correlated to piston shifts occurring for N2 (see FIG. 3-A). Similarly, the piston motions of the two C-helices are not correlated.

Changes in the four inter-monomer tilt angles are strongly correlated to each other, as explained in FIG. S3 and FIG. S4 in Text S1. We can therefore describe changes in the four inter-monomeric tilt angles by only one inter-monomer tilt angle 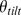, which describes the tilt angle between the helices of monomer 1 and of monomer 2. To determine whether other motions are related to the piston shift observed for states 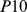 and 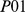, we plotted two-dimensional probability plots as a function of the N2 and C2 piston shifts and the tilt angle 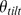 (FIG. 3-B). There is no difference in monomer tilt angle for the native conformation and the piston-shifted conformations 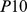 and 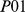, because 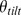 is between 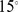 and 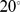 for all states.

We investigated the rotation 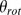 of helix N1 with the piston shift of the same helix in FIG. 3-C. For the piston shift, two minima occur, which have similar values for the rotational angle. Clearly, a piston shift seems to be uncorrelated to either changes in tilt or rotation of the helices. In FIG. 3-A5, we plotted the negative log probability distribution of the rotation of helices N1 and C2. This contour plot shows only one minimum and a small positive correlation, which seems in contrast with FIG. 2-C. This figure shows one relaxation process, whereas FIG. 2-A5 shows the average relaxation to either 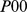 or 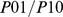.

### Metadynamics simulations along tilt and rotation yield a single free energy minimum

Although the WT* MD simulations occasionally visit a novel conformation, they only sample a small part of the conformational space and are inherently out of equilibrium. To explore the equilibrium behavior we enhanced sampling by applying adaptive biasing potentials in the MD simulations, in the well-tempered metadynamics approach [23], [24]. As the biasing potentials are based on predefined collective variables (CVs), described in the Methods section, the approach allows the identification of important CVs in conformational transitions.

First, we performed a metadynamics simulation, biasing the inter-monomer tilt angle 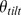. In the first attempt, the range of 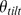 was unlimited, resulting in values for 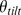 of 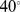 and higher. At such a large tilt angle, the hydrophobic core is disrupted, leading to dissociation of the complex. Once the complex has fallen apart, it is impossible to return to the intact state using only the inter-monomer tilt angle as a CV. To prevent these severe changes we constrained the range of 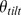 by adding a repulsive wall at 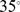. Note that HAMP embedded in a sensory protein complex is very unlikely to explore very large tilt angles.

**Figure 5:**
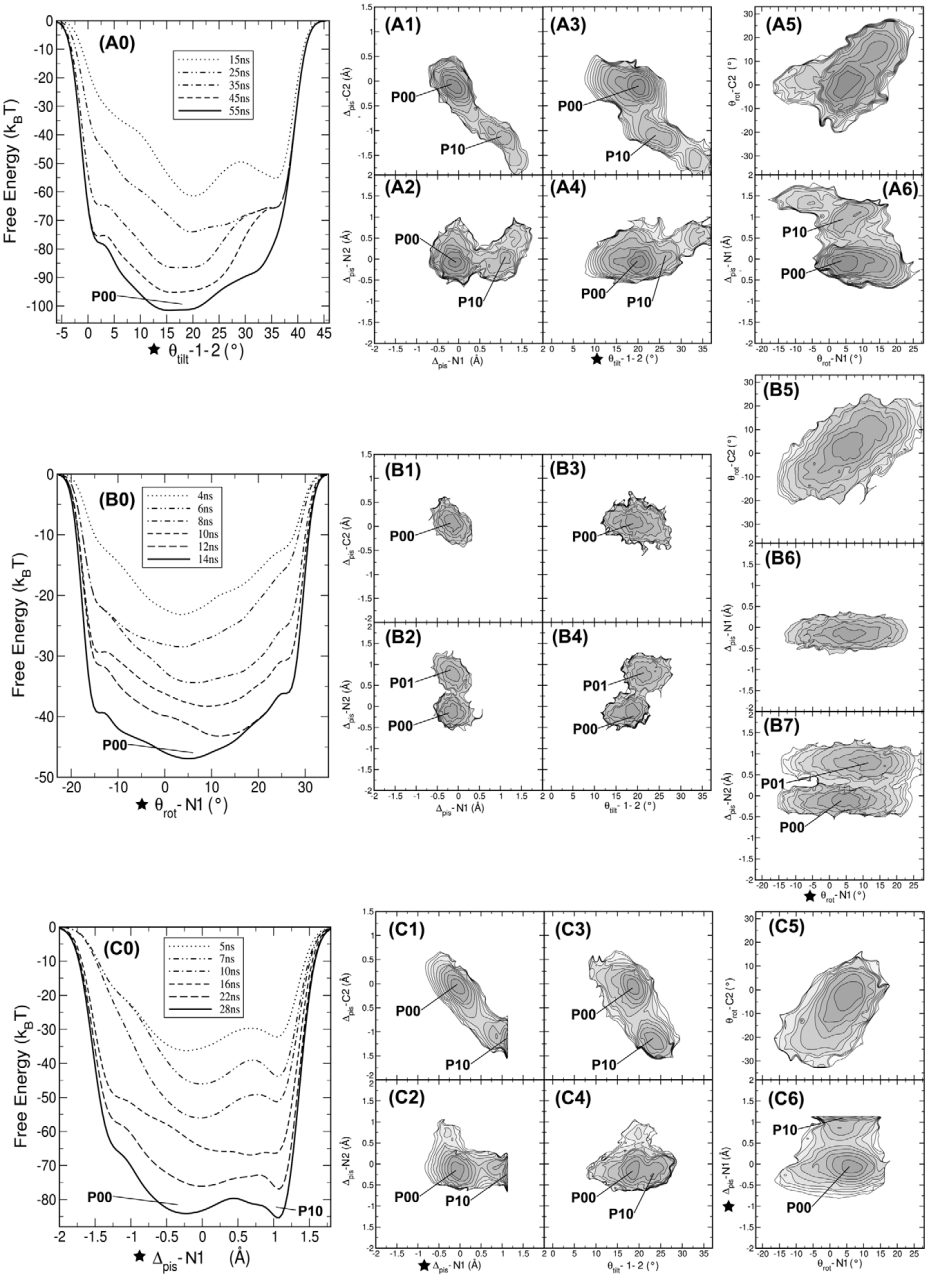
One-dimensional bias on tilt angle, piston shift and rotation angle. In the metadynamics simulations the biasing potential was applied to (A) Intermonomer tilt angle 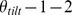 (B) rotation angle 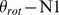 (C) piston shift 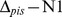. For each simulation the free energy evolution is given in panels A0,B0,C0, with the reweighted free energy profiles in (A1,B1,C1) 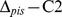 versus 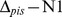; (A2,B2,C2) 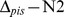 versus 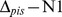; (A3,B3,C3) 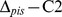 versus 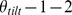; (A4,B4,C4) 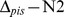 versus 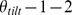; (A5,B5,C5) 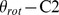 versus 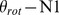; (A6,B6,C6) 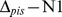 versus 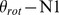. Stars indicate the CV on which the bias was applied. Labels indicate stable states. Contour lines are rendered every 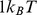. The metadynamics simulations show that biasing the piston shift reveals an additional conformational state and that the piston and tilt motions are correlated.

FIG. 5-A0 shows the time evolution of the biasing potential along 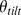. After 35 ns, the shape of the profile does not change anymore. At this point the negative biasing potential represents the free energy profile along 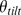 and shows one broad free energy minimum. The width of the minimum is consistent with the results from the conventional MD simulations. Even though the biasing potential acts on one CV, we can obtain the free energy surface along other CVs by using a reweighting procedure [25]. The resulting profiles are shown in FIG. 5-A1–A6. The patterns of piston motions as observed in the WT* simulations are partially reproduced. Only the pair of helices N1-C2 exhibits piston motions, while 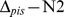 does not change (see FIG. 5-A1,A2). When 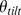 reaches 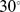, 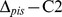 becomes more negative, see FIG. 5-A3 (accordingly 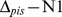 reaches 1.5 Å, see FIG. 5-A1). This shows that even though we bias the inter-monomer tilt angle, a spontaneous transition to the 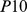 state can occur as well. The reweighted free energy surface as a function of 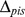 and 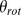 for N1 in FIG. 5-A6 does not show such correlated motions.

Hulko et al. [3] postulated a mechanism, called the gearbox model, for relaying signals in HAMP via concerted rotation of the helices, thereby changing the packing of the hydrophobic layers. Using metadynamics we can test this mechanism by biasing the rotation of one helix and observe the rotation of the other helices. We performed a one-dimensional metadynamics simulation using 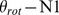 as the CV. The first attempt, in which the rotational angle was completely unconstrained, resulted in unfolding of the helix. We therefore applied constraints to the range of 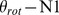, with a lower boundary at 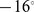 and an upper boundary at 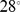. Recent structural studies revealed that concerted rotation does occur, but that the range is different per hydrophobic layer [7], [19]. This means that biasing the rigid body rotation of the entire helix will inevitably lead to unfolding, as some parts of the helices rotate differently than others.

FIG. 5-B0 shows the time evolution of the biasing potential and the resulting free energy surface. From 6 ns onwards, the profile changes very little and reveals one broad minimum centered at 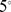, consistent with the observations for the conventional MD simulations of the wild type NMR structure (see TAB. 1). The reweighted free energy surface in FIG. 5-B5 reveals a positive correlation between 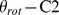 and 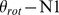, on which the bias was applied, similar to that observed in the WT* simulations. FIG. 5-B1–B4 show that during this metadynamics simulation, not only the native state 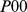 is visited, but also a piston-shifted state, 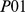 with only one transition 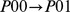 and one transition backwards. This transition is not the result of the biasing potential on 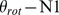, but a spontaneous fluctuation in the piston mode of pair C1 and N2. This is revealed by the free energy surface as a function of 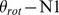 and 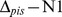, in which 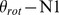 is one broad minimum, completely uncorrelated to the changes in 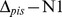, see FIG. 5-B7.

### Biasing the piston motion reveals additional conformational states

The WT* simulations revealed that Af1503 HAMP can adopt different conformations, which can be distinguished by the piston shift. In the metadynamics simulations biasing the rotation and tilting these two conformations do not show up in the profile of the biasing potential, whereas they do appear spontaneously in the reweighted free energy surface. If these states are truly metastable, a metadynamics simulation biasing the piston motion should in principle reveal them most efficiently. We therefore performed a one-dimensional well-tempered metadynamics simulation on 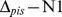. To prevent unfolding of the helices, we constrained the range of the piston shift to 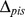 = −1.4 Å as a lower bound and 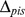 = 1.2 Å as the upper bound. The resulting free energy profile is shown in FIG. 5-C0 and shows two free energy minima. One minimum is located at 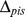 = −0.25 Å and corresponds to the native conformation of the wild type, the 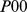 state. The other minimum is located at 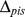 = 1.1 Å and corresponds to the 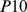 state. FIG. 4 shows a representative conformation of the 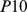 state.

The reweighted free energy surface as a function of 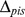 of N1 and C2 in FIG. 5-C1,C2 demonstrates that correlated piston shifts occurred only for the pair that contains the helix on which the bias was applied. The other pair did not undergo piston motions. FIG. 5-C3 shows the free energy profile as a function of the inter-monomer tilt angle and the piston shifts. An increase in the tilt angle of 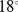 to 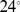 is correlated with an increase in the piston shift of C2 from 0 Å to −1.1 Å. The free energy surface as a function of the helical rotation angle shows again that the change in rotation is uncorrelated to the change in piston, and furthermore that the change in rotation in N1 is positively correlated with the change in 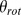 for C2.

**Figure 6:**
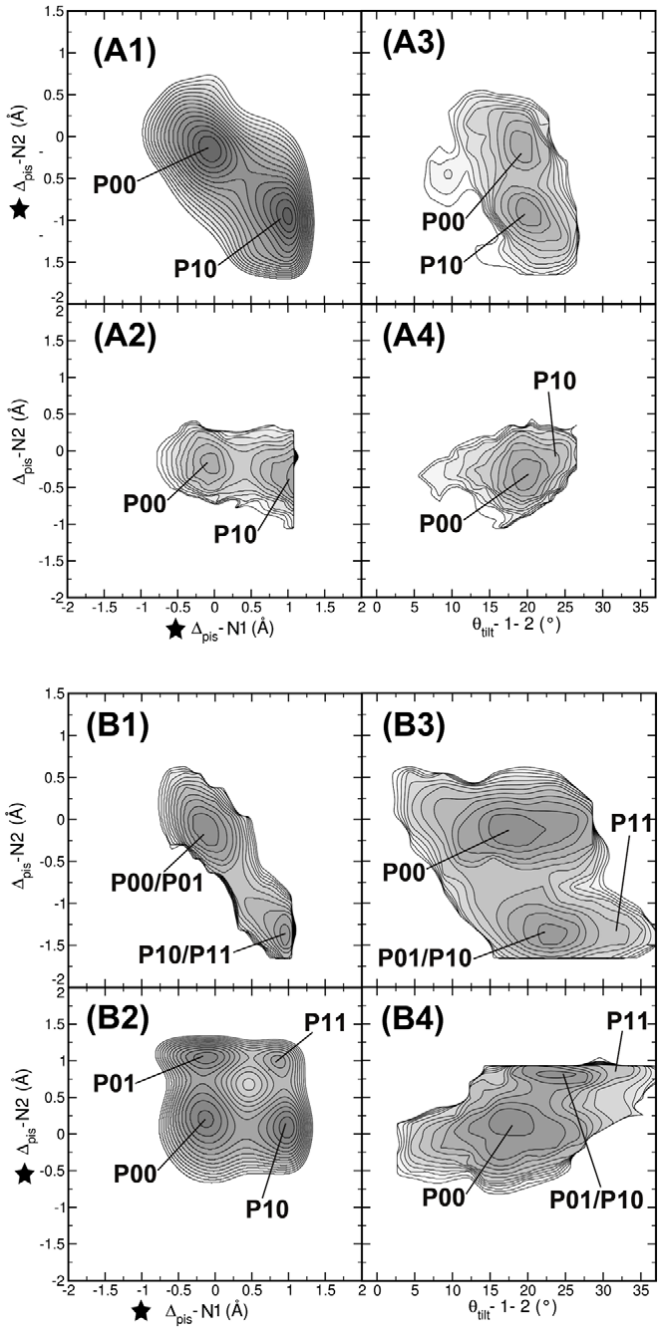
Two dimensional bias on piston motions. In the metadynamics simulations the biasing potential was applied to (A) 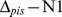 and 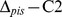; (B) 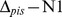 and 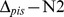. The results are shown as free energy profiles in the following projections: (A1,B1) 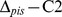 versus 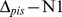; (A2,B2) 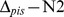 versus 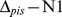; (A3,B3) 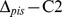 versus 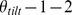; (A4,B4) 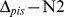 versus 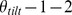. (A1) and (B2) show the free energy landscapes of biased CVs, the other subplots are corresponding reweighted free energy surfaces. Stars indicate the CV on which the bias was applied. Labels indicate stable states. Contour lines are rendered every 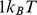. The two-dimensional metadynamics simulations show that the piston motions of helices N1 and C2 are oppositely correlated, and that this correlation also exists for the C1,N2 pair.

We have found a negative correlation between the piston shifts of the N-terminal helix of one monomer and the C-terminal helix of the other monomer, (see FIG. 5-C1). To investigate this correlation further, we performed a two-dimensional metadynamics run, biasing both 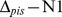 and 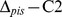. FIG. 6-A1 shows the resulting two-dimensional free energy surface. The profile reveals two minima that are very similar to the states 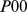 and 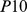 identified in the conventional MD study and the one-dimensional metadynamics simulation biasing a single piston shift. The piston shift of the N1-helix is strongly anti-correlated with the piston shift of the C2-helix. In FIG. 6-A2, the reweighted free energy profile as a function of 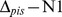 and 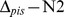 shows that piston shifts in this helical pair are not correlated, as no change occurs for 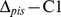, when 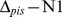 shows a piston shift (see FIG. 6-A3, A4).

Biasing the piston shift of one helix resulted in enhanced piston shift of only one other helix, which has to be part of the other monomer and be at the other end of the protein chain. We performed a two-dimensional metadynamics simulation on 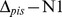 and 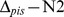 to further investigate whether piston motions between N-terminal helices are truly not correlated. The resulting free energy profile is shown in FIG. 6-B2 and reveals four minima. Three minima, 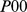, 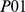 and 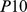 have also been observed as well in the conventional MD study and represent respectively states in which no piston shift has occurred, a piston shift has occurred in the N1-C2 pair, and a piston shift has occurred in the N2-C1 pair. In addition, this free energy surface contains an extra minimum 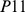 at (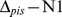 = 1 Å, 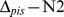 = 1 Å) in which both helical pairs have undergone a piston shift.

Biasing one helical pair does not result in the inter-monomer tilt angle changing in a concerted way with the changes in piston shift. In FIG. 6-A3,A4, for 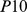 or 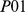, the inter-monomer tilt angle 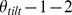 rests at 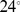. The occurrence of the 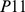 state goes hand-in-hand with an increase of the inter-monomer tilt angle 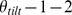 from 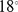 of WT to 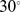 (see FIG. 6-B3, B4). This shows that, although we bias on piston shifts, changes in the inter-monomer tilt angle occur spontaneously.

To further explore the 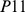 state, we performed MD simulations of this piston-shifted state, see FIG. S5 in Text S1. The 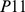 state is only meta-stable, as it returns to either the 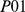 state or the 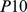 state within nanoseconds.

## Discussion

Our molecular dynamics simulations show that the structure of wild type Af1503-HAMP is very stable at room temperature, whereas the simulations of the A291F mutant show that the NMR structure of this variant is not at all stable at room temperature. For A291F-HAMP, we found that the system either shows loss of helical structure or can relax to a conformation similar to A291F-HAMP fused to a DHp domain [19]. The structure of the A291F mutant was suggested as an alternative conformation for HAMP, as increasing the volume of the hydrophobic sidechain at position 291 would change the packing in the hydrophobic core from complementary 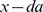 to 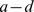. If the 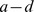 packing would truly be an additional metastable state for HAMP, this structure would be as stable as the one assumed by wild-type Af1503-HAMP. However, our simulations showed that this structure relaxes either to the native conformation, or to a conformation 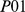 or 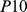 with an upward piston shift of the N-terminal helix in one monomer and a downward piston shift of the C-terminal helix in the other monomer.

The metadynamics simulations aimed at exploring the equilibrium free energy landscape of HAMP revealed an additional stable state when the piston shift was biased. This additional metastable state shows a strong similarity to the piston-shifted state found in the WT* simulations. In this state, the N-terminal helix of one monomer moves up, and the C-terminal helix of the other monomer moves down. Cysteine crosslinking studies of the HAMP domain in the Aer sensor protein, which senses the intracellular redox potential, resulted in a similar observation, in which a correlation between the N-terminal helix of one monomer and the C-terminal helix of the other monomer is observed [17]. The metadynamics simulations sampled this correlated motion for both symmetry related pairs. Moreover, the two-dimensional metadynamics simulation that biased both correlated motions uncovered the existence of an additional state in which both helical pairs in the piston shifted conformation. These piston-shifted states also show an increased inter-monomer tilt angle. Biasing directly on the tilt collective variable resulted in the spontaneous sampling of the 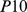 state, showing that the piston shift and change in tilt angle are strongly coupled.

**Figure 7:**
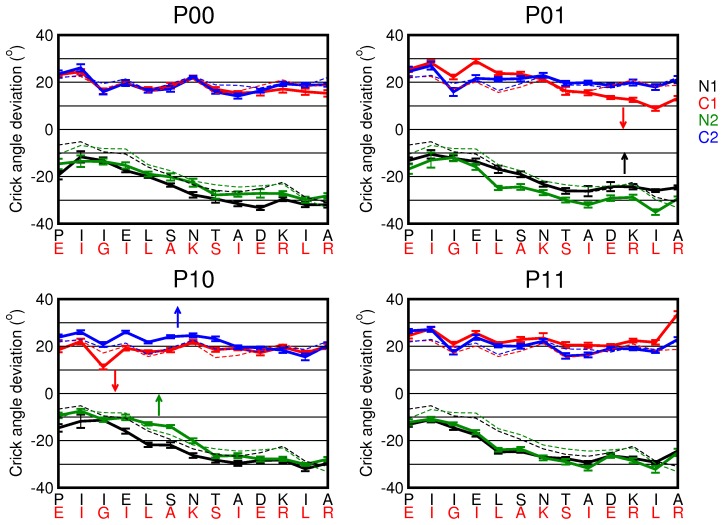
Deviation of the ideal Crick angles for the four piston states. The Crick angle deviation is plotted as a function of residue in the N and C-helices for the 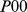, 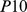, 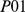 and 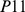 states. The error bars indicate the variation over 40 snapshots. The dashed lines represent the Crick angle deviations as measured for the NMR structure of wild-type Af1503-HAMP (PDB-code 2L7I). There are little differences between the states.

The role of helical rotation is less clear, as our results seem to indicate that changes in rotation occur independently with respect to changes in the piston shifts or tilt angles. Directly biasing the rotation angle results in small changes of the other rotation angles, but not in visiting an additional conformational state. As we calculated the rotation as a single value for an entire helix, we cannot directly compare our results with the gearbox model. To this end, we extracted 40 snapshots from each piston-shifted state and computed several properties related to four-helical bundles for each residue, using the SamCC software [4], [7]. One of these properties is the Crick angle per residue, measured as the angle between the center of the bundle, the 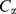-atom of the residue and the point on the principal axis of the helix closest to the residue. The Crick angles are known for an ideal helical bundle, so we can also measure the deviation from the ideal 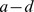 packing. In FIG. 7 we show the deviation from the ideal Crick angle for all four piston states. In all states the overall deviation is close to 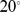 for the N-helices and 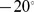 for the C-helices and does not come close to zero in any of the states. The curves representing the helices in the 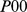 state are very similar to the curve measured for the NMR structure of wild-type Af1503-HAMP (dashed lines). The four states exhibit small differences in the Crick angle deviation, as indicated by the arrows.

By using metadynamics simulations, we have attempted to obtain an estimate of the barriers separating the different states in HAMP. In these simulations, we encountered the problem that the collective variables required to correctly describe such a transition are complex. The free energy landscapes that we have obtained are therefore projected and underestimate the true barrier, which could be obtained when biasing along the perfect reaction coordinate. Finding a better reaction coordinate requires the use of transition path sampling [26], [27] or path-metadynamics [28].

In FIG. 4, we summarized the piston-shifted states the isolated HAMP domain can visit. 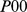 exhibits no piston shift and has an inter-monomer tilt angle 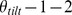 of 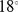. In the 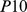 state, helix N1 moves upwards, and helix C2 moves downward. Also, the tilt angle increases to 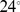. The tilt angle is the same in the 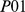 state, but in this state, symmetrically related to the 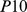 state, helix N2 moves upwards, and helix C1 moves downward. State 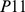 results when both helical pairs move and has a monomer tilt angle of 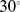. MD simulations on the 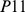 piston shifted state show that the 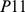 conformation is only meta-stable, as it relaxes to either the 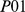 or the 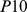 state within nanoseconds.

A typical chemotaxis receptor, which has two ligand binding sites, can, in principle, visit three states, unbound, half bound and fully bound. Such states could coincide with the conformational states we observed for HAMP. The asymmetric 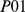 and 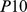 states would then represent the half-bound receptor configuration, whereas the unbound state would be represented by either the 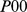 or the 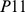 state. Several investigations have provided evidence that the transmembrane helices perform an inward piston motion upon ligand binding [14]–[16]. Based on our observations, we speculate that HAMP can perform an inward piston motion by starting in the 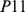 state and converting to either the 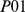 or the 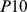 state, implying that the ligand-free state of the receptor corresponds to the 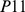 state. Consequently, the 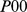 state would then represent the fully-bound state. Replacing the original HAMP domain in a chemotaxis fusion protein by Af1503-HAMP resulted in a consistently activating response for all constructs examined [7], [19]. Because we observed the piston-induced states only by activating Af1503-HAMP, either by starting from a different conformation in the WT* simulations or by adding adaptive biasing potentials in the metadynamics simulations, Af1503-HAMP in the fusion protein must be in the 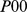 state and, as a consequence, will relay an activating signal.

On the other hand, extensive mutations on the HAMP domain of Tsr have lead to the suggestion that HAMP functions as a dynamic bundle [12], [13] implying that the Tsr-HAMP domain is flexible and can adopt multiple conformational states. Possibly, these could be the different conformational states we observed in this work and will be the topic for future investigations.

## Methods

### Molecular dynamics setup

In all simulations we used the GROMACS software package, version 4.0.7 [29] in combination with the OPLS all atom force field [30]. As starting structures for the MD simulations we used the NMR structure of the wild-type Af1503-HAMP domain from *A*. fulgidus, PDB entry 2ASW [3] (superseded by 2L7H [7]), and the A291F mutant (PDB entry 2L7I) [7]. These structures are shown in (FIG. 1). All systems were solvated in a periodic cubic box with dimension 67 Å. All systems were filled with 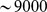 TIP4P water molecules [31], followed by the removal of water molecules that overlap with protein atoms or reside in a hydrophobic location isolated from the bulk. NaCl was added by replacing water molecules by Na+ and Cl− ions at random. 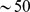 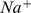 and 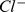 ions were added to mimic physiological conditions at [NaCl] = 0.2M and maintain electrostatic neutrality of the system.

All systems were energy minimized using the conjugate gradient method. To equilibrate the hydrogen atoms and water molecules the heavy atoms in the protein were position-restrained during 10 ps of molecular dynamics at a temperature of 298 K and a pressure of 1 bar. The van der Waals interaction cut-off radius was 11 Å. Electrostatic interactions beyond a cut-off of 11 Å were treated with the Particle Mesh Ewald method [32], [33] using a grid spacing of 0.12 nm. All bonds were constrained using LINCS [34], allowing for a time step of 2 fs. Temperature was kept constant using the Nosé-Hoover thermostat [35], and pressure was kept constant using the Parrinello-Rahman barostat [36]. For sampling, we performed 8 independent, 4 of 40 ns and 4 of 60 ns runs for WT Af1503, 3 55 ns runs for A291F, and 24 50 ns runs are obtained for the A291F structure with WT sequence, each starting with velocities randomly drawn from a Maxwell-Boltzmann distribution at 298 K. All simulations were performed in parallel on an IBM pSeries 575 supercomputer.

### Analysis of MD

We calculated several properties of HAMP, including the helical root mean square deviation (RMSD), and the number of helical hydrogen bonds 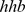. We calculated the helical RMSD with respect to three experimentally resolved structures: (I) 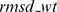 for the NMR structure of the wild type HAMP, pdb entry 2ASW; (II) 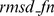 for the A291F mutant, the NMR structure, pdb entry 2L7I, and (III) 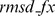 for A291F, its crystal structure, pdb entry 3ZRV. As the terminal residues 276–281 and 327–403 exhibit relatively large fluctuations, these residues were excluded from the RMSD calculations. Linker residues between N and C (297–311) were also excluded to ensure that the RMSD reflects only the structural differences in the helices. Therefore 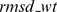, 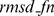 and 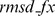 were calculated only for the residues with observable helical structure throughout the simulations, which constitute residues 282–296 from N and residues 312–326 from C (FIG. 1). The number of helical hydrogen bonds 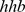 was computed between all residue pairs 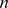 and 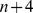. A helical hydrogen bond is counted if the distance between the backbone oxygen atom of residue 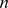 and the backbone nitrogen atom of residue 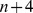 is smaller than 3.5 Å and the angle between the acceptor, donor and hydrogen is smaller than 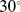.

We also measure three helical motions, described by models in literature explaining the mechanism of signal relay by HAMP: (I) the rotation of a helix, (II) tilt angles between two helices and (III) piston motion of the helices. These properties are measured via the collective variables defined in the PLUMED package [37], as described below.

### Metadynamics

**Figure 8:**
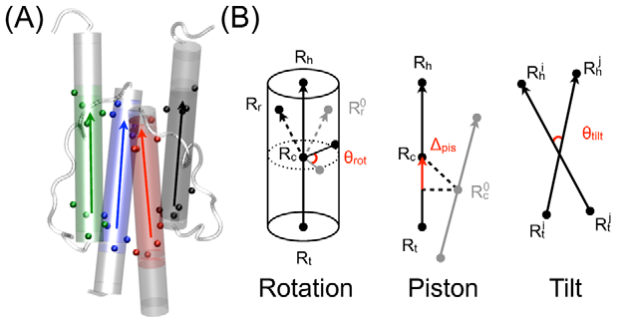
Definition of helical motions. (A) Positions of four consecutive 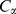 atoms, indicated by colored spheres (*black* N1; *red* C1; *green* N2; *blue* C2) are used to define the head and tail groups. The arrow points at the head. (B) Three collective variables (CVs) describing helical motions: rotation 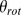, piston shift 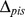 and tilt 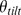. Reference vectors are indicated in gray. The CV definitions are indicated in *red*. See main text for an explanation of the definitions.

Metadynamics is an enhanced sampling method that performs history-dependent sampling in a reduced collective variable (CV) space [23]. We encoded three new CVs in PLUMED [37], a package that contains the metadynamics algorithm for the tilt angle, the helical rotation and the piston shift. Rotation of a helix and tilt angles between two helices are measured via the CV 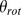 and 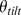 respectively. Piston motions of a single helix are quantified through the CV 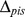. As the CV 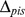 and 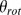 involve a reference structure, we must align the reference structure with respect to the current frame throughout the simulation. We used the NMR strcture of wild-type Af1503-HAMP as a reference, PDB-code 2ASW. These CVs can only be used under the condition that the helices under study are relatively stable without severe bending or twisting. We tested our implementation of these three new CVs by comparing the analytically calculated values of the derivative of the CVs implemented in PLUMED and those of a numerically computed derivative via a very small change in the CVs. FIG. 8 shows a schematic representation of the definitions for the three CVs.

As each turn of a helix consists of four consecutive residues, we define a vector 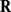 representing each helix based on four consecutive 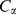 atoms at head and tail of the helix. Let 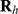 and 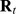 be the head and tail, then 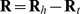.

#### Piston

We define a CV piston that measures the movement of a vector along itself. In short, this is a projection of the vector connecting the center of mass of a vector 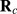 and the reference vector 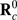 onto the vector 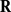 itself. Note that this reference structure is first aligned with the current coordinates to remove overall translation and rotation. Let 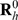 and 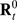 represent the initial positions of the head and tail of the vector 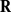, we have
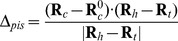
(1)For HAMP, we use four consecutive 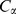 atoms at each of a helix to define the head and the tail (FIG. 8-A).

#### Rotation

We defined the rotation of a helix along its own axis using three groups of atoms: the head group 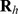, the tail group 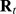 and the rotational reference group 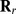. This rotational reference group is defined as the center of mass of a group of atoms, such that 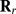 is not on the principal axis of a helix. The vector 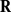 is defined by the difference between the head and tail 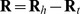. The center of mass of the vector is then 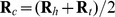. With 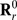 the rotational reference group of a reference structure, we compute 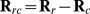 and 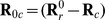. Using 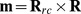 and 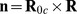, rotation is then defined by
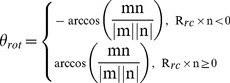
(2)In our setup, 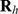 and 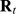 are defined as the center of mass of four consecutive 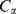 atoms at each end of a helix. 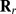 is defined by the center of mass of a series of 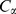 atoms separated by three 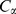 atoms, e.g. 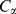 of residue 283, 287, 291 and 295 for N, inside the hydrophobic core and 313, 317, 321 and 325 for C, outside on the solvent exposed surface. Note that the reference structure is first aligned with the current coordinates to remove the overall translational and rotational motions

#### Tilt angle

The tilt angle between two vectors 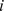 and 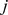 can be expressed as follows:
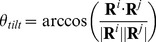
(3)The definition of the head and tail of a helix is similar to those defined for 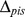 and 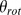. For the monomer tilting angle between monomers 1 and 2, we let 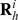, 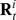 encompass all head and tail 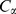 atoms from monomer 1 while 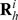, 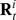 include all corresponding 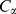 atoms from monomer 2.

We used the well-tempered approach in our metadynamics setup [24]. In well-tempered metadynamics, the Gaussian height 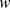 is automatically rescaled during the simulation:
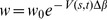
(4)where 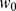 is the initial Gaussian height, 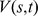 the instantaneous biasing potential, 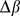 a parameter with the dimension of the inverse temperature: 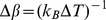 and 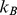 the Boltzmann constant. This equation ensures the convergence of the 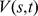 as follows
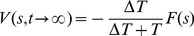
(5)where 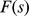 is the free energy surface of CV 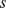, 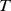 the temperature of the system and 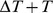 the (fictitious) CV temperature. 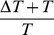 is referred to as the bias factor. A proper choice of the bias factor enables one to tune the extent of exploration on the energy scale for a metadynamics simulation.

All well-tempered metadynamics runs are performed using the same settings as described in the MD setup. The bias factors used in the one-dimensional, two-dimensional and four-dimensional well-tempered metadynamics simulations are 40, 10 and 30 respectively. The free energy surfaces of the biased CVs are generated via summing up the hills distributed along the selected CV space. Widths of the hills for an angle CV (rotation or tilt) and a piston CV are chosen 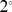 and 0.2 Å respectively. Gaussian hills are deposited every 2 ps with a height of 0.25 kJ/mol for one-dimensional and two-dimensional metadynamics simulations and 0.3 kJ/mol for the four-dimensional metadynamics biasing all inter-monomer tilt angles. Using larger hills than the chosen values results in loss of accuracy of the free energy profile and unfolds the helices within a few ns.

To prevent unnecessary sampling outside the CV region of interest, we applied potential walls in the following form:
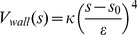
(6)where 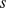 is the value of CV, 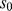 is the position of the wall, 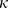 an force constant and 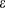 a rescaling factor. This potential is only active when 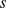 is larger than the upper bound or smaller than the lower bound. 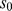 is set as follows (I) lower bounded at 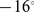 and upper bounded at 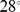 for 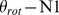; (II) 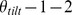 upper bounded at 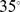; (III) lower bound at −1.4 Å and upper bound at 1.2 Å for 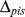 of N1 and C1. 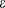 is set 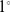, 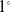 and 0.1 Å for rotation, tilt, and piston shift respectively. 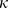 is 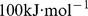 for rotation or tilt angles and 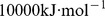 for piston.

Another advantage of well-tempered metadynamics is the possibility to use the efficient reweighting algorithm that allows the computation of the free energy surfaces of other properties than the biased one [25]. In well-tempered metadynamics, as the simulation proceeds, the bias potential 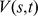 evolves more and more adiabatically, i.e. the system becomes more and more in instantaneous equilibrium under the action of its internal potential and 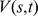). If one assumes such adiabatic evolution of 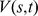 and let 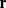 be the configurational coordinate, one has
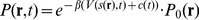
(7)where 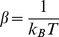, 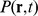 and 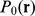 are the biased and canonical distribution respectively, with
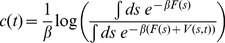
(8)defined as the time-dependent bias offset. The biased distribution at 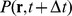 can be expressed as:
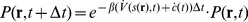
(9)In Ref [25], by realizing
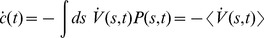
(10)where 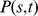 is the CV probability distribution in the biased ensemble, Bonomi et al established the following expression of 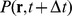


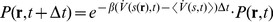
(11)With this expression of the variation of the biased distribution in terms of the variation of the bias potential, it is possible to obtain 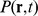, and thus the free energy surfaces of other unbiased CV(s) in this biased ensemble, along the progression of 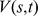.

## Supporting Information

Text S1The supporting information Text S1 contains four sections. The first section describes the results obtained from Molecular Dynamics simulations of the A219F variant. The second section contains a figure displaying all possible piston combinations for the WT* simulations. The third section describes the correlation between the intermonomeric helical tilt angles and in the fourth section we investigated the stability of the 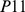 state.(PDF)
