# Supplementary material for: The HAMP Signal Relay Domain Adopts Multiple Conformational States through Collective Piston and Tilt Motions
Source: PLoS Comput Biol. 2013 Feb 28;9(2):e1002913. doi: 10.1371/journal.pcbi.1002913 (PMC3585426; doi:10.1371/journal.pcbi.1002913)
Supplement: Text S1 — The supporting information Text S1 contains four sections. The first section describes the results obtained from Molecular Dynamics simulations of the A219F variant. The second section contains a figure displaying all possible piston combinations for the WT* simulations. The third section describes the correlation between the intermonomeric helical tilt angles and in the fourth section we investigated the stability of the state. (PDF) [file pcbi.1002913.s001.pdf]

Supporting Information

Lizhe Zhu[1], Peter G. Bolhuis[1], Jocelyne Vreede[1,*],
1 Van 't Hoff Institute for Molecular Sciences, University of Amsterdam, Science Park 904 1098 XH, Amsterdam, The Netherlands
∗ E-mail: J.Vreede@uva.nl

The Supplementary Information accompanying *The signal relay HAMP domain adopts multiple conformational states through collective piston and tilt motions* contains four sections. The first section describes the results obtained from Molecular Dynamics simulations of the A219F variant. The second section contains a figure displaying all possible piston combinations for the WT* simulations. The third section describes the correlation between the intermonomeric helical tilt angles and in the fourth section we investigated the stability of the $P11$ state.

The mutant A291F is not stable in solution.

Using the NMR structure of the A291F mutant as a starting point (PDB code 2L7I), we performed three MD simulations of 55 ns. Visual inspection of the trajectories shows that in two out of three, the helices bend at the second layer, when orienting the HAMP domain with N-termini upward. In one trajectory the domain stays intact, and shows remarkable agreement with the crystal structure resolved for the A291F mutant, fused to a DHp domain (PDB code 3ZRV). This becomes apparent when plotting probability plots for these three trajectories as a function of the number of helical hydrogen bonds and $rmsd\_fx$ the RMSD with respect to the crystal structure of the fused domain. FIG. 1-A shows two minima, one at (hhb=51, $rmsd\_fx$=0.6 Å), representing the structure close to the crystal structure, and another at (hhb=47, $rmsd$=1.7 Å), indicating the conformations with loss of helical character. Taking only the simulation in which the helices do not bend in consideration, we computed $rmsd\_wt$ and $rmsd\_fn$, with $rmsd\_fn$ the RMSD computed against the NMR structure of the A291F mutant (PDB entry 2L7I). The resulting probability plot shown in FIG. 1-B exhibits one minimum at ($rmsd\_fn$ = 1.4 - 2.1 Å, $rmsd\_wt$ = 1.0 - 1.6 Å), indicating that this structure relaxes to conformations different from the NMR structures of WT and A291F. It is surprising that the conformation resolved by NMR for this mutant, indicated by the star in FIG. 1-B, is not stable at all in our MD simulation set up. In solution, this mutant exists as a mixture of parallel and anti-parallel conformations. The crystal structure of this mutant in isolation revealed an anti-parallel complex. Only when fused to another protein a crystal structure of the parallel form could be resolved. It is therefore no surprise that this mutant conformation shows large flexibility in our simulations.

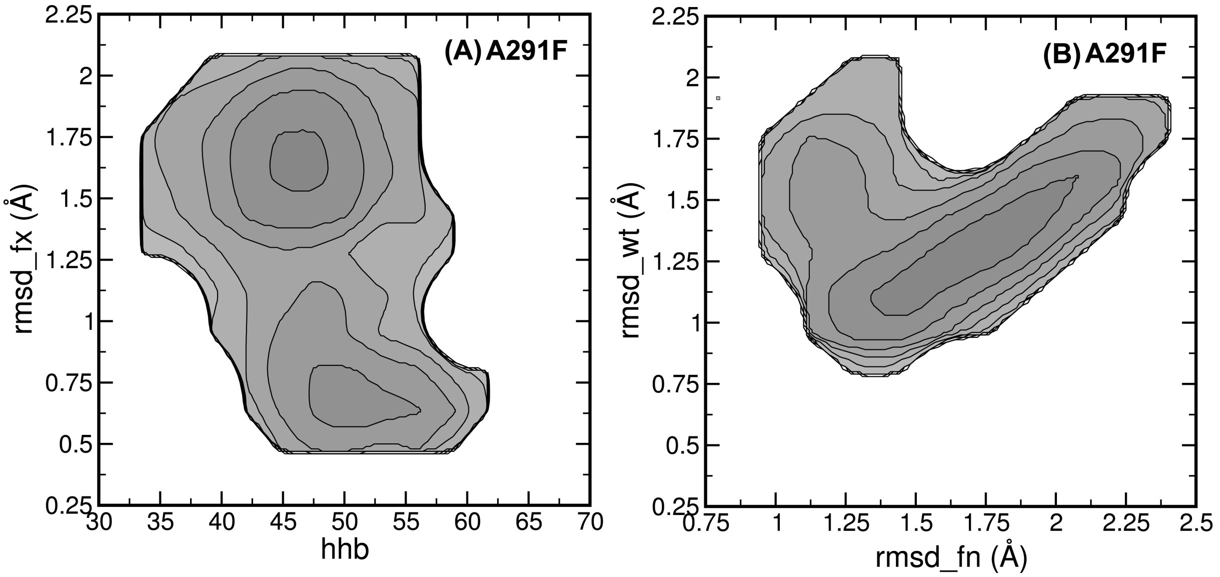

Figure 1. MD of A291F system. The negative log of the probability distributions are shown for the A291F system as (A) a function of the number of helical hydrogen bonds *hhb* and the RMSD with respect to the NMR structure of A291X in a crystal structure of a fusion protein (PDB-code), *rmsd_fx* and (B) as a function of the RMSD with respect to the NMR structure of wild-type 1503-HAMP, *rmsd_wt*, and the RMSD with respect to the NMR structure of A291F, *rmsd_fn*.

Piston shifts in WT*

FIG. 2 shows all possible combinations of the piston motions in the WT* simulations. The labels $P00$ indicate the conformations close to WT and typically have a piston shift close to zero. The states $P10$ and $P01$ are symmetrically related and involve a correlated piston shift of helices N1 and C2 for $P10$ (reffigb2wt:pis-C) or helices C1 and N2 for $P01$ (reffigb2wt:pis-D). Furthermore, the N-terminal helices N1 and N2 move up with respect to state $P00$ whereas the C-terminal helices C1 and C2 move downward.

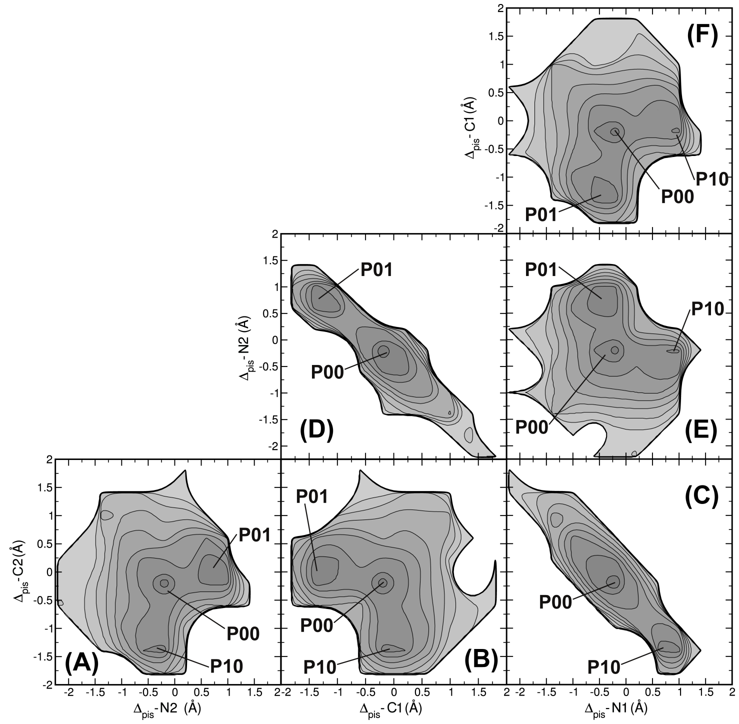

Figure 2. Piston modes in WT* trajectories. The negative log of the probability distributions are shown for the WT* system as a function of the piston shifts (A) Δ_{pis}-C2 versus Δ_{pis}-N2; (B) Δ_{pis}-C2 versus Δ_{pis}-C1; (C) Δ_{pis}-C2 versus Δ_{pis}-N1; (D) Δ_{pis}-N2 versus Δ_{pis}-C1; (E) Δ_{pis}-N2 versus Δ_{pis}-N1 ; (F) Δ_{pis}-C1 versus Δ_{pis}-N1. The labels indicate the states $P00$, $P01$ and $P10$. Contour lines are rendered every $1k_BT$.

Inter-monomeric tilt angle

We measured the four inter-monomeric tilting angles θ_{tilt}-N1-N2, θ_{tilt}-N1-C2, θ_{tilt}-C1-N2 and θ_{tilt}-C1-C2 for the WT* simulations, resulting in the negative log probability plots shown in FIG. 3. The plots all have a single minimum at values close to 20°. However, the shape of the profiles shows that if θ_{tilt}-N1-N2 changes, the three other inter-monomeric tilt angles change as well.

Since we observed strong correlations among all four inter-monomer tilt angles, we performed a 30ns four-dimensional metadynamics simulation biasing on all four inter-monomer tilt angles. FIG. 4 shows two-dimensional projections of the resulting free energy profile (FES) and reveal that the strong correlations are reproduced in this four-dimensional metadynamics simulation.

It is therefore possible to reduce the description of the inter-monomeric tilt angles by using only a single tilt angle that describes the tilt between monomers θ_{tilt}-M1-M2.

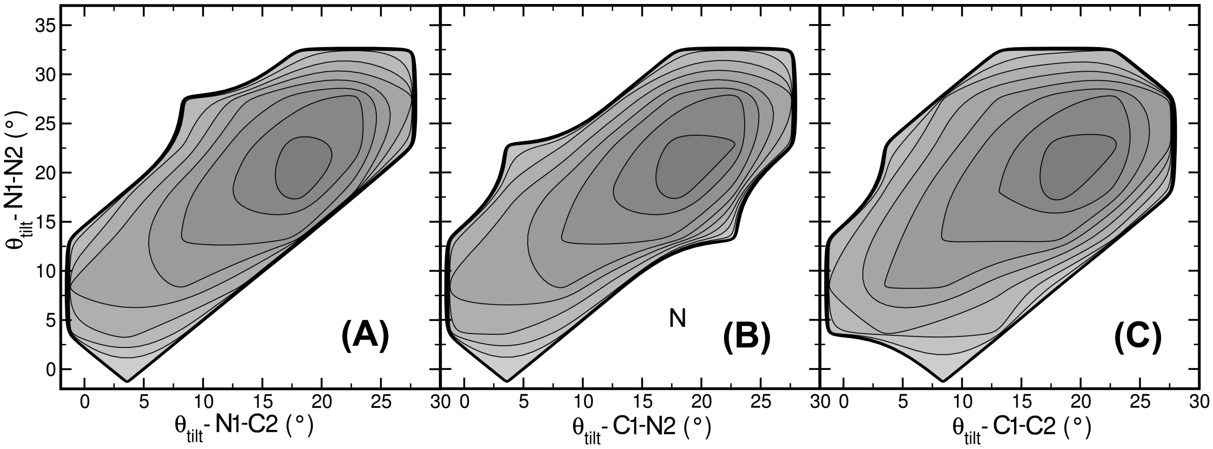

Figure 3. inter-monomer tilt angles in WT* trajectories. The negative log of the probability distributions are shown for the WT* system as a function of the tilt angles θ_{tilt}-N1-N2 (vertical axis) and on the horizontal axis (A) θ_{tilt}-N1-C2; (B) θ_{tilt}-C1-N2; (C) θ_{tilt}-C1-C2. Contour lines are rendered every $1k_BT$.

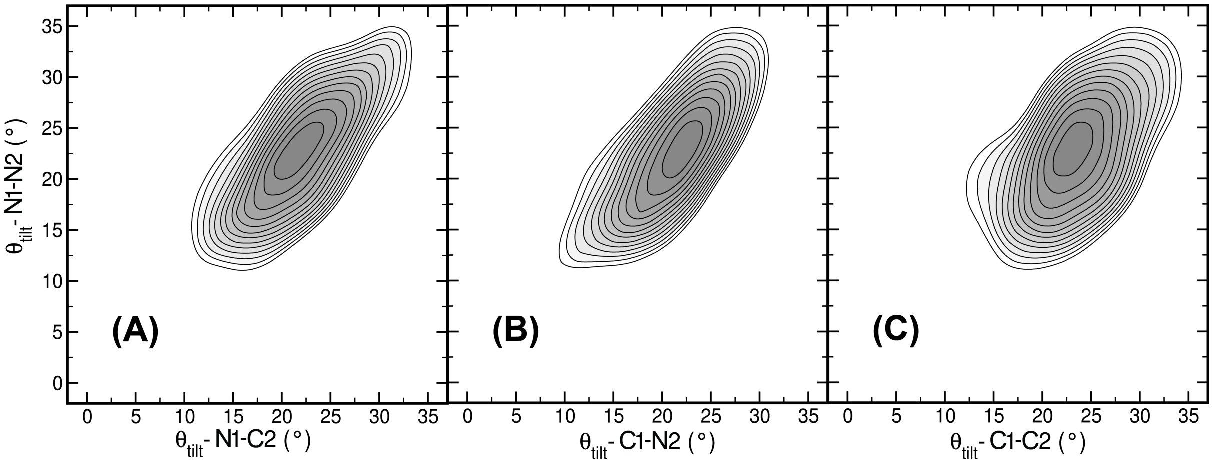

Figure 4. Four-dimensional bias on all four inter-monomer tilt angles. Free energy projection in two-dimensional representation: (A) θ_{tilt}-N1-N2 versus θ_{tilt}-N1-C2; (B) θ_{tilt}-N1-N2 versus θ_{tilt}-C1-N2; (C) θ_{tilt}-N1-N2 versus θ_{tilt}-C1-C2. Contour lines are rendered every $1k_BT$.

Stability of the P11 state

To investigate the stability of the $P11$ piston shifted state, we extracted nine conformations from the 2D metadynamics simulation on the N-terminal piston shifts and performed 14 MD simulations in total starting with different velocities. FIG. 5 shows the time traces of the piston shifts. All simulations start with pistons shifts of 1 Å for the N-terminal helices and -1 Å for the C-terminal helices. Within nanoseconds the system relaxes to either the $P01$ state of the $P10$ state, as indicated by the piston shifts of respectively the N1-C2 pair or the N2-C1 pair changing to 0 Å. This means that the $P11$ state is only meta-stable.

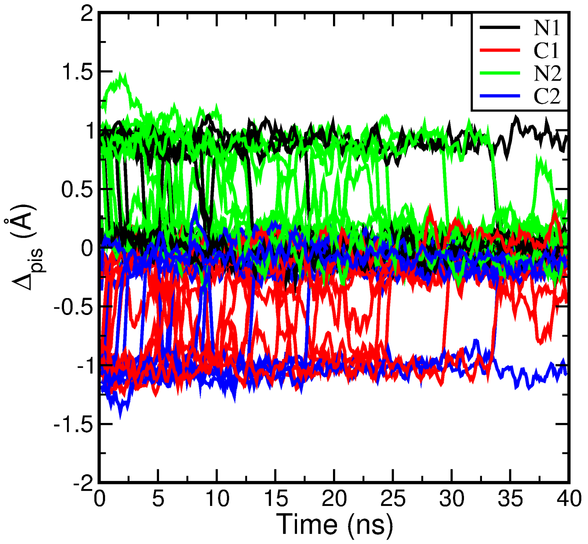

Figure 5. Stability of the piston-shifted states. Time traces of the piston shifts for the four helices in HAMP for the $P01$ state.
